# Golgi fragmentation in amyotrophic lateral sclerosis, an overview of possible triggers and consequences

**DOI:** 10.3389/fnins.2015.00400

**Published:** 2015-10-27

**Authors:** Vinod Sundaramoorthy, Jessica M. Sultana, Julie D. Atkin

**Affiliations:** ^1^Department of Biomedical Sciences, Faculty of Medicine and Health Sciences, Macquarie University SydneySydney, NSW, Australia; ^2^Department of Biochemistry and Genetics, La Trobe Institute for Molecular Science, La Trobe UniversityMelbourne, VIC, Australia

**Keywords:** amyotrophic lateral sclerosis, Golgi fragmentation, ER stress, axonal degeneration, secretory trafficking inhibition, autophagy dysfunction

## Abstract

Amyotrophic Lateral Sclerosis (ALS) is an invariably fatal neurodegenerative disorder, which specifically targets motor neurons in the brain, brain stem and spinal cord. Whilst the etiology of ALS remains unknown, fragmentation of the Golgi apparatus is detected in ALS patient motor neurons and in animal/cellular disease models. The Golgi is a highly dynamic organelle that acts as a dispatching station for the vesicular transport of secretory/transmembrane proteins. It also mediates autophagy and maintains endoplasmic reticulum (ER) and axonal homeostasis. Both the trigger for Golgi fragmentation and the functional consequences of a fragmented Golgi apparatus in ALS remain unclear. However, recent evidence has highlighted defects in vesicular trafficking as a pathogenic mechanism in ALS. This review summarizes the evidence describing Golgi fragmentation in ALS, with possible links to other disease processes including cellular trafficking, ER stress, defective autophagy, and axonal degeneration.

## Introduction

The Golgi apparatus (referred to as “Golgi” hereafter) acts as a dispatching station whereby proteins and lipids newly synthesized in the ER are transported to the endosomal system, secretory granules, or plasma membrane. In spite of being a highly dynamic organelle (Griffiths et al., 1989), the Golgi normally maintains a characteristic morphology, consisting of flattened membrane stacks known as cisternae, and associated vesicles. The stacks of Golgi cisternae are interconnected laterally by tubules, forming a ribbon-like network (Rambourg and Clermont, 1990; Polishchuk and Mironov, 2004), usually in the perinuclear region of the cell, adjacent to the centrosome (Linstedt, 2004). The Golgi comprises of three functional compartments: the cis-Golgi, which being the nearest compartment to the ER, forms the entry face to the Golgi, the medial-Golgi, which is responsible for the modification, sorting and packaging of proteins for transportation, and finally the trans Golgi network, which forms the exit face of the Golgi (Rothman and Wieland, 1996; Glick and Nakano, 2009). Specific types of intracellular vesicles are associated with the Golgi. Secretory protein cargo buds from the ER via coat protein complex II (COPII) coated vesicles, to form tubulovesicular structures known as the ER-Golgi intermediate compartment (ERGIC), which eventually fuse with the cis-Golgi (Appenzeller-Herzog and Hauri, 2006). In contrast, the reticular trans-Golgi network (TGN) produces clathrin-coated vesicles which are targeted to endosomes and secretory vesicles in specialized cell types such as neurons (De Matteis and Luini, 2008). In addition to secretory trafficking, the Golgi is also responsible for the post-translational modification of proteins and lipids, including glycosylation (Stanley, 2011), sulfation (Baeuerle and Huttner, 1987), and proteolytic cleavage (Xu and Shields, 1993).

## The Golgi in neurons

**Figure 1:**
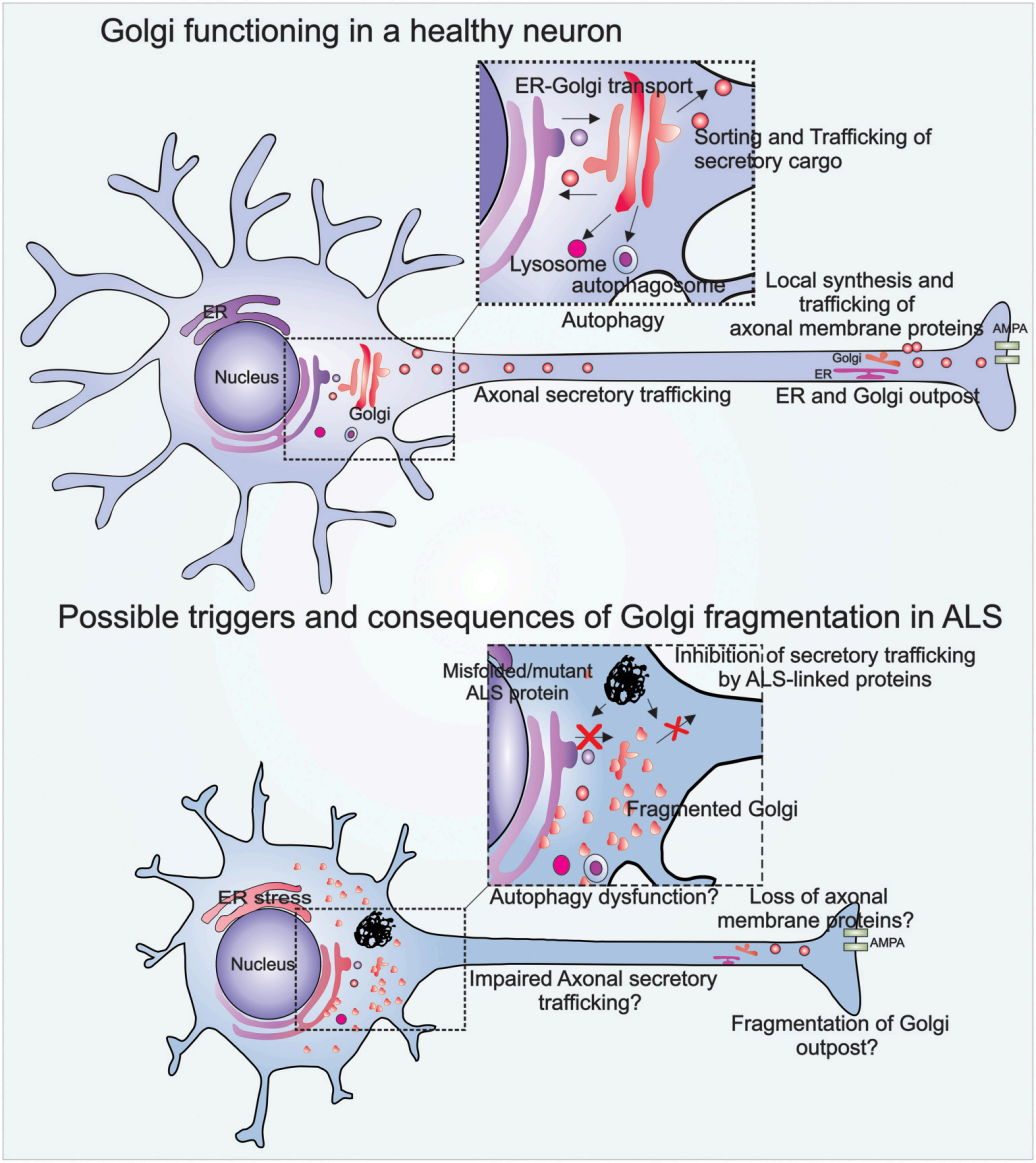
**Illustration of Golgi functions in a healthy neuron, and Golgi fragmentation in an ALS-affected neuron**. The Golgi in a healthy neuron regulates vesicular trafficking from the ER to the plasma membrane. The Golgi is also involved in the biogenesis of autophagosomes and lysosomes. Golgi outposts in healthy axons are involved in local synthesis and trafficking of axonal membrane proteins. Golgi fragmentation in ALS may be triggered by pathogenic mutant proteins that inhibit vesicular trafficking between the ER-Golgi, and Golgi to plasma membrane. Possible consequences of Golgi fragmentation in ALS include autophagy dysfunction, impaired axonal secretory trafficking, and loss of axonal homeostasis.

Neurons are highly specialized cells with unique functional and morphological characteristics. Interestingly, in neurons the Golgi forms specialized “Golgi outposts” localized in axons and dendrites, which are discrete structures that are discontinuous from the somatic Golgi (Figure 1). These Golgi outposts are not fully characterized, but are thought to facilitate local secretory trafficking within neurites (Horton and Ehlers, 2003; Merianda et al., 2009). Axonal transport is an important property in neurons which involves trafficking of cellular proteins and vesicles within the axon, towards or away from the cell body. The relationship between axonal transport and transport within the soma is not fully understood, but these processes are clearly linked and involve the Golgi (Hirokawa and Takemura, 2005; Schwarz, 2013).

## Fragmentation of the Golgi apparatus

The Golgi is capable of undergoing profound morphological changes during normal cellular processes such as mitosis, as well as in pathological conditions. These morphological changes result in disruption of its characteristic ribbon-like network, forming a fragmented Golgi. Golgi fragmentation during mitosis facilitates equal distribution of the Golgi into the resulting daughter cells (Sütterlin et al., 2002). However, irreversible Golgi fragmentation occurs in pathological situations, when apoptosis is activated. Under these conditions, structural proteins within the Golgi are cleaved by the action of caspases (Lane et al., 2002). The Golgi also fragments when vesicular secretory trafficking is perturbed (Dascher and Balch, 1994; Wilson et al., 1994), which may also occur in pathological conditions. The morphological changes evident during fragmentation of the Golgi are attributed to two possibilities, either the Golgi membranes break into smaller dispersed vesicular structures (Figure 1), or the Golgi fuses with the ER upon fragmentation, which is then recycled, and it remerges at ER exit sites, dispersed throughout the cytoplasm (Cole et al., 1996; Storrie et al., 1998; Pelletier et al., 2000; Glick, 2002).

Golgi pathology is a feature of neurodegenerative diseases including Alzheimer's disease (Sun et al., 2008), Parkinson's disease (Fujita et al., 2006), Creutzfeldt-Jakob disease (Sakurai et al., 2000), multiple system atrophy (Sakurai et al., 2002), and ALS. Interestingly, Golgi fragmentation is often detected as an early event in these conditions, prior to apoptosis (Gosavi et al., 2002; Liazoghli et al., 2005; Atkin et al., 2014; van Dis et al., 2014), suggesting that Golgi fragmentation could be a trigger for neurodegeneration rather than a simple consequence of neuronal death. We review here the evidence describing Golgi fragmentation in ALS, and discuss recent studies implicating impairment of ER-Golgi mediated vesicular trafficking as a possible trigger. We also predict possible downstream consequences of Golgi fragmentation in ALS, and we examine links to other pathologies, including ER stress, autophagy dysfunction, and axonal degeneration.

## Amyotrophic lateral sclerosis

Whilst 90% of ALS cases are sporadic, 10% of cases are familial, caused by mutations in genes encoding ubiquitously expressed proteins, including transactive response DNA binding protein (TDP-43), fused in sarcoma (FUS), optineurin, superoxide dismutase 1 (SOD1), and Chromosome 9 open reading frame 72 (C9orf72) (Renton et al., 2014) (Table 1). Although, the etiology of ALS remains unknown, RNA dysfunction and disruption to proteostasis are widely implicated as pathogenic mechanisms (Ling et al., 2013). Dysfunction to proteostasis includes protein misfolding and aggregation, ER stress, Golgi fragmentation, autophagy dysfunction, inhibition of cellular trafficking, and axonal degeneration. Like other neurodegenerative disorders, a pathological hallmark of ALS is the accumulation of intracellular inclusions containing misfolded protein aggregates (Wood et al., 2003; Blokhuis et al., 2013). Interestingly, wildtype (WT) forms of TDP-43, FUS, optineurin, and SOD1 may be recruited into ubiquitinated protein inclusions in sporadic ALS patients (Neumann et al., 2006; Deng et al., 2010; Blokhuis et al., 2013). Cytoplasmic accumulation, hyperphosphorylation and/or aggregation of TDP-43 is present in almost all cases of ALS (approximately 97%) (Ling et al., 2013). Transgenic mice overexpressing mutant SOD1^G93A^ are the most widely used animal models of disease, which recapitulate many clinical and pathological features of ALS (Gurney et al., 1994). Increasing evidence now links ALS to frontotemporal dementia (FTD), with recent studies suggesting that ALS and FTD represent opposite ends of the disease spectrum (Ling et al., 2013).

**Table 1:** **List of major ALS genes and ALS-linked proteins with established intracellular and axonal trafficking functions**.

**Major ALS genes**	**Chromosome location**	**Disease feature**	**Percentage of ALS**		**References**
			**Familial**	**Sporadic**	
C9ORF72	9p21	ALS, FTD, ALS with FTD	40	7	DeJesus-Hernandez et al., 2011; Renton et al., 2011
SOD1	21q22	ALS	12	1–2	Rosen et al., 1993
TARDBP	1p36	ALS, FTD, ALS with FTD	4	1	Sreedharan et al., 2008
FUS	16p11	ALS, FTD, ALS with FTD	4	1	Kwiatkowski et al., 2009; Vance et al., 2009
**ALS-linked proteins**	**Cellular trafficking function**				
C9ORF72	Possible involvement in Rab-mediated membrane trafficking processes. Predicted to function as a Rab guanine nucleotide exchange factor (GEF).				Zhang et al., 2012; Levine et al., 2013; Farg et al., 2014
Optineurin	Vesicular trafficking of secretory proteins, autophagosomes and lysosomes.				Sahlender et al., 2005; Tumbarello et al., 2012; Sundaramoorthy et al., 2015
VCP	Secretory protein trafficking.				Ballar et al., 2011; Arita et al., 2012; Yi et al., 2012
	Translocation of misfolded proteins from the ER to cytoplasm for proteosome degradation.				
Profilin1	Polymerization of the actin cytoskeleton.				Pantaloni and Carlier, 1993
VAPB	Vesicular ER-Golgi trafficking, dendritic membrane protein trafficking. Also involved in microtubule organization.				Skehel et al., 2000; Kuijpers et al., 2013
SQSTM1/p62	Mediates AMPA receptor trafficking at the synapse and and also dynein-linked cellular trafficking.				Jiang et al., 2009; Calderilla-Barbosa et al., 2014
	Mediates Cargo recognition and trafficking in autophagy.				
Alsin	Functions as a GEF for Rab5 and Rac1.			
	Involved in in Rab5-endocytic trafficking and Rac1 regulation of the actin cytoskeleton.				Otomo et al., 2003; Topp et al., 2004
CHMP2B	Component of endosomal sorting complex required for transport-III (ESCRT-III), essential for endocytic trafficking.				Urwin et al., 2010
Dynactin	Mediates dynein and kinesin 2 driven intracellular and axonal trafficking on microtubules.				Schroer, 2004; Ross et al., 2008
Neurofilament heavy chain	Essential for maintaining axon structure and function.				Liu et al., 2004
TUBA4A	Component of microtubule cytoskeleton.				Oakley, 2000
Peripherin	Type III neuronal intermediate filament involved in peripheral axon outgrowth and regeneration.				Oblinger et al., 1989
Spatacsin	Involved in axonal anterograde vesicular trafficking.				Pérez-Brangulí et al., 2014
Phosphoinositide 5 phosphatase	Associated with late endosome/lysosomal membrane trafficking pathways.				Chow et al., 2007; Ruscher and Wieloch, 2015
Sigma-1 receptor	Involved in lipid rafts associated transport of proteins and lipids to plasma membrane.				Pabba et al., 2014; Ruscher and Wieloch, 2015

Defects in intracellular trafficking, particularly within the axon, are implicated in ALS (Bilsland et al., 2010; Ikenaka et al., 2012; Alami et al., 2014). The “dying back” or slow degeneration of distal to proximal axons is associated with loss of motor neurons in ALS (Fischer et al., 2004; Dadon-Nachum et al., 2011; Moloney et al., 2014). In SOD1 mice, distal axonopathy and denervation of neuromuscular junctions are observed prior to the onset of clinical manifestations (Fischer et al., 2004). Fast-fatigable motor neurons with the longest axons and highest metabolic demands, are the most susceptible to axonal degeneration (Frey et al., 2000; Fischer et al., 2004). It has been suggested that lack of supply of essential proteins and lipids to distal axons is associated with axonal degeneration (Perlson et al., 2010).

Autophagy is an important proteostatic mechanism to degrade misfolded proteins in post-mitotic neurons (Thomas et al., 2013). It is therefore not surprising that defects in autophagy are present in ALS, however the nature of autophagy defects in ALS remains unclear. Autophagosomes accumulate in ALS patient brain tissues (Sasaki, 2011), implying that both induction of autophagy and inhibition of clearance of autophagsomes exist in ALS. However, more recent studies have demonstrated that formation of the autophagosome is impaired in cells expressing ALS mutant FU (Soo et al., 2015b) and in cells with reduced C9orf72 expression (Farg et al., 2014). Furthermore, mutations in proteins involved in endosomal sorting and trafficking which are required for the formation of autophagosomes (VCP, p62, dynactin, and RAB7) are also associated with ALS (Otomo et al., 2012). Activation of the unfolded protein response (UPR) and ER stress are well-documented pathogenic features in human ALS patients (Ilieva et al., 2007; Atkin et al., 2008; Oyanagi et al., 2008; Walker et al., 2010) and in animal/cellular disease models associated with mutant FUS, TDP-43, C9orf72, optineurin, and SOD1 (Atkin et al., 2006; Oh et al., 2008; Walker and Atkin, 2011; Farg et al., 2012; Walker et al., 2013; Zhang et al., 2014; Sundaramoorthy et al., 2015). Interestingly ER stress develops first in the most vulnerable motor neurons in SOD1^G93A^ mice, 60 days before disease onset (Saxena et al., 2009), thus implicating ER stress as an active mechanism inducing cell death in ALS. Similarly, Golgi fragmentation is a prominent pathological feature in human ALS, and appears at a similar time point in SOD1 mice models (Gonatas et al., 1992; Mourelatos et al., 1994; van Dis et al., 2014).

## Golgi fragmentation in ALS

Fragmentation of the Golgi was first identified in ALS patient motor neurons over 20 years ago (Gonatas et al., 1992). In contrast to control patients, the Golgi in ALS patients was reduced and fragmented, appearing as disconnected punctate structures, similar to its morphology in cells treated with microtubule depolymerisation agents (Mourelatos et al., 1990; Gonatas et al., 1992). Since then, other studies have confirmed Golgi fragmentation in 10–50% sporadic patients (Gonatas et al., 2006; van Dis et al., 2014) and up to 70% of familial ALS patient motor neurons, bearing SOD1, FUS or optineurin mutations (Fujita et al., 2008; Ito et al., 2011). Interestingly, Golgi fragmentation is more prominent in larger human motor neurons, such as those in the cerebral cortex (Fujita et al., 1999) and anterior horn (Fujita et al., 2000), suggesting they are specifically vulnerable to disturbances in Golgi function.

Golgi fragmentation is also present in spinal anterior horn cells in sporadic ALS patients with cytoplasmic mislocalization of WT TDP-43, implying that a link exists between TDP-43 and Golgi pathologies (Fujita et al., 2008). Similarly, Golgi fragmentation is present in transgenic rats expressing mutant TDP-43^M337V^ (Tong et al., 2012), in mutant SOD1^G93A^ transgenic mice and in neuronal cells expressing SOD1^G93A, G85R^ mutants (Mourelatos et al., 1996; Stieber et al., 2004). Interestingly, Golgi fragmentation precedes SOD1 inclusion formation, neuromuscular denervation, and mitochondrial-mediated apoptosis in low-copy number SOD1^G93A^ transgenic mice, implying it is upstream in pathogenesis (van Dis et al., 2014). Similarly, ALS patients with optineurin mutations (<1% familial cases) demonstrate Golgi fragmentation in ~70% of anterior horn cells (Ito et al., 2011). Furthermore, Golgi fragmentation is present in cells expressing ALS-linked mutant FUS, optineurin and vesicle-associated membrane protein B (VAPB) (Teuling et al., 2007; Farg et al., 2013; Sundaramoorthy et al., 2015). However, despite being widely associated with ALS, the cellular events triggering Golgi fragmentation and the resulting consequences are not established. Increasing evidence implicates inhibition of vesicular trafficking between the ER-Golgi in ALS, which may explain the previous observations of Golgi fragmentation.

## Impairment of cellular trafficking is a trigger for Golgi fragmentation in ALS

The ER-Golgi compartments form the first part of the cellular secretory pathway, hence they are sensitive to alterations in the rate of trafficking (Pelletier et al., 2000; Lee et al., 2004), and trafficking inhibition leads to dysfunction in both compartments. In the ER, accumulation of unfolded nascent secretory proteins in the lumen triggers the UPR. The UPR initially aims to reduce protein synthesis and increase protein folding (Graves et al., 2001; Preston et al., 2009). However, when impairment of trafficking persists, prolonged UPR results in activation of apoptosis (Szegezdi et al., 2006; Hetz, 2012). Similarly the organization of the Golgi depends on efficient bidirectional vesicular transport with the ER (Nassif et al., 2010). The formation of Golgi stacks requires continuous recycling of Golgi proteins to/from the ER (Lippincott-Schwartz et al., 2000). Inhibition of protein export from the ER disrupts Golgi organization (Storrie et al., 1998), resulting in the formation of tubulovesicular Golgi clusters, some of which fuse with the ER (Puri and Linstedt, 2003), which can further increase ER stress. Similarly, inhibition of vesicular trafficking from the Golgi to plasma membrane leads to protein accumulation within the Golgi. If prolonged, this can fragment the Golgi (Persson et al., 1992; Zolov and Lupashin, 2005; Zhou et al., 2013). Approximately one-third of the human proteome transverses through the Golgi destined for transmembrane, synaptic, axonal, or extracellular locations (Braakman and Bulleid, 2011). Hence disruption to intracellular trafficking involving the Golgi could severely compromise neuronal function and viability.

We recently demonstrated that Golgi-associated vesicular trafficking is inhibited in cells expressing ALS-mutant proteins: SOD1, FUS, TDP-43, and optineurin, providing an intriguing mechanism explaining Golgi fragmentation in patient tissues (Sundaramoorthy et al., 2013, 2015; Atkin et al., 2014; Soo et al., 2015a). Furthermore, inhibition of ER-Golgi transport by mutant SOD1 preceded all other cellular pathologies examined in neuronal cells, including ER stress, Golgi fragmentation, protein aggregation, inclusion formation, and apoptosis (Atkin et al., 2014). This implies that ER-Golgi trafficking defects may trigger ER-Golgi pathology in SOD1-ALS cases. More recently we demonstrated that mutant forms of both FUS and TDP-43 impair the incorporation of secretory cargo into COPII vesicles budding off from the ER, impeding protein export from the ER, while mutant SOD1 was shown to inhibit ERGIC-Golgi trafficking by destabilizing microtubules (Soo et al., 2015a). Furthermore, we have also demonstrated that misfolded WT SOD1 also impairs ER-Golgi trafficking similar to mutant SOD1, resulting in ER stress and Golgi fragmentation (Sundaramoorthy et al., 2015), although it remains controversial whether misfolded WT SOD1 is present in sporadic ALS tissues (Liu et al., 2009; Forsberg et al., 2010; Grad et al., 2014). However, it is tempting to speculate that impairment of ER-Golgi trafficking is a common trigger for Golgi fragmentation in sporadic and familial ALS. Similarly, we have also shown that expression of ALS-optineurin mutants impair myosin VI-mediated protein trafficking from the Golgi to plasma membrane, also inducing Golgi fragmentation (Sundaramoorthy et al., 2015). Hence these results imply that impairment of distinct protein trafficking pathways by different ALS-linked proteins are specific triggers for Golgi fragmentation in ALS (Figure 1).

Consistent with this notion, mutations in genes encoding proteins directly involved in intracellular trafficking are present in familial ALS (Table 1). Firstly, mutations in the gene encoding the p150^Glued^ subunit of the dynein/dynactin complex were reported in sporadic and familial ALS (Münch et al., 2004). The ALS causing mutation impedes binding of p150^Glued^ to microtubules, resulting in dysfunctional dynein/dynactin-mediated transport (Levy et al., 2006). Similarly, mutations in proteins directly involved in the ER-Golgi secretory pathway, including VAPB and VCP, are present in ALS (Nishimura et al., 2004; Johnson et al., 2010; Yi et al., 2012; Kuijpers et al., 2013). Recent findings of mutations in ALS-associated genes that encode cytoskeletal associated proteins provide additional evidence for trafficking disruption in ALS. Mutations in profilin 1, which mediates the conversion of soluble G-actin to functional F-actin, (Wu et al., 2012), and in tubulin alpha 4A (TUBA4A), a component of microtubules, were recently reported in familial ALS (Smith et al., 2014).

The identification of hexanucleotide (GGGGCC) repeat expansion mutations in *C9ORF72* as the major cause of familial ALS and FTD (40%), further links cellular trafficking to ALS. Whilst the normal cellular function of C9orf72 was initially unknown, bioinformatics studies first predicted that C9orf72 functions in Rab-mediated trafficking (Zhang et al., 2012; Levine et al., 2013). Rab proteins form a large family of small guanosine triphosphate (GTP)ases that regulate vesicular trafficking at distinct cellular membranes (Stenmark and Olkkonen, 2001). Rab proteins are activated by conversion from an inactive guanosine diphosphate (GDP)-bound state to an active GTP-bound form, which is catalyzed by guanine nucleotide exchange factors (GEFs) (Stenmark and Olkkonen, 2001; Cherfils and Zeghouf, 2013). Bioinformatics predicted that C9orf72 functions as a RabGEF, because of the strong sequence and structural similarity to other evolutionary conserved differentially expressed in normal and neoplastic cells (DENN) domain-containing RabGEFs (Zhang et al., 2012; Levine et al., 2013). Consistent with these predictions, we demonstrated that C9orf72 associates with multiple Rabs including Rab1, which mediates ER-Golgi transport (Farg et al., 2014). Furthermore, we also found that depletion of C9orf72 using siRNA impaired autophagy and endocytic trafficking from the plasma membrane to Golgi (Farg et al., 2014). Whilst the hexanucleotide repeat expansion is present within an intronic region of *C9ORF72*, expression of C9orf72 protein is reduced in ALS patients causing haploinsufficiency (DeJesus-Hernandez et al., 2011; Haeusler et al., 2014). Hence this would disrupt the normal trafficking function of C9orf72 in ALS. However, recent studies have argued against this mechanism of pathogenesis (Koppers et al., 2015). Nevertheless, we also demonstrated increased association of C9orf72 with Rab7 and Rab11 in C9orf72-ALS patients, implying that intracellular trafficking is dysregulated in C9orf72-ALS, although the mechanism remains unclear (Farg et al., 2014). However, further studies are required to examine the relationship between C9orf72 and trafficking defects, including whether the Golgi is fragmented in C9orf72-ALS patients.

## Golgi fragmentation and autophagy dysfunction

The initial step in autophagy is the formation of a double-membraned phagophore, which then expands in size, engulfing defective proteins, damaged cellular organelles, or pathogens, forming the autophagosome (Reggiori and Klionsky, 2005). Although the membranes forming the autophagosome originate from multiple cellular organelles, the ER is implicated as the primary source of membrane because the omegasome, the autophagosome precursor, originates from ER cisternae (Hayashi-Nishino et al., 2009). However, the Golgi is necessary for subsequent autophagosome elongation, and Golgi-mediated trafficking provides membrane components for autophagosome biogenesis. Beclin1, which is located in the trans-Golgi network, recruits other autophagy-related (Atg) proteins for assembly into the autophagosome, (Kihara et al., 2001) and Atg9-positive vesicles cycle from the Golgi to deliver membranes to the developing autophagosome (Young et al., 2006; Webber et al., 2007). Blocking ER to cis-Golgi transport, or transport from the trans-Golgi to plasma membrane/endosomes, reduces autophagosome formation in mammalian cells (Zoppino et al., 2010; Guo et al., 2012). Furthermore, the Golgi recognizes and sorts lysosomal enzymes, which are then packaged into vesicles that bud from the trans-Golgi, forming lysosomes (Griffiths et al., 1988; Kornfeld and Mellman, 1989; Riederer et al., 1994). Hence these observations imply that disruption of Golgi-associated trafficking may impair autophagosome formation.

In contrast, fragmentation of the Golgi has also been shown to increase autophagosome biogenesis by feeding Atg9-positive fragmented Golgi membranes during starvation-induced autophagy (Takahashi et al., 2011). Pharmacological induction of Golgi fragmentation with Brefeldin A or Golgicide increases autophagosome biogenesis and induces accumulation of autophagosomes (Naydenov et al., 2012), but it can also block autophagosome formation in some cases (Nishida et al., 2009). Observations of Golgi fragmentation and autophagy dysfunction imply a possible link between these two pathologies in ALS. Hence examination of the pathological relationship between Golgi fragmentation and autophagy in ALS is warranted.

## Golgi fragmentation and axonal homeostasis

Motor neurons differ from other neurons in that they are exceptionally large, with long axons, up to 1 m in length in an adult human. These distal axons require membrane and cytoskeletal proteins, neurotransmitter receptors, and lipids to maintain synaptic plasticity, synaptogenesis, excitability, dendritic, and neurite outgrowth (Horton and Ehlers, 2004; Tuck and Cavalli, 2010; Ori-McKenney et al., 2012). These components must be transported from the ER/Golgi in the cell body over long distances along the axon. In addition to this traditional route, proteins are also synthesized via axonal ribosomes (Koenig et al., 2000; Kun et al., 2007) and mRNA (Taylor et al., 2009; Jung et al., 2012), thus facilitating local protein synthesis. Proteins synthesized in neurites are processed and secreted via Golgi outposts (Horton and Ehlers, 2003; Merianda et al., 2009) (Figure 1). These Golgi outposts share similar molecular markers to the somatic Golgi (Gardiol et al., 1999; Horton and Ehlers, 2003) and they handle secretion of essential axonal/dendritic membrane proteins (Lu et al., 2001; Passafaro et al., 2001). Similarly, cytoskeletal proteins processed via Golgi outposts are essential for axonal regeneration and plasticity of dendritic spines (Matus, 2000; Gu et al., 2008; Tuck and Cavalli, 2010; Shirao and González−Billault, 2013). Fragmentation of the neuronal Golgi would therefore be expected to impair normal axonal functions. In support of this notion, induction of Golgi fragmentation with Brefeldin A reduces synaptic potentiation and AMPA receptor expression on the postsynaptic membrane (Broutman and Baudry, 2001), and reduces axonal outgrowth (Jareb and Banker, 1997). Interestingly, fragmentation of somatic and dendritic Golgi in motor neurons accompanied by trafficking defects, preceded axonal retraction and muscle denervation in mice models of ALS (van Dis et al., 2014). Therefore, Golgi fragmentation may be an important trigger for loss of axonal homeostasis and degeneration of motor neurons in ALS.

## Conclusion

An emerging concept in ALS is that the diverse mechanisms implicated in pathology are inter-linked, and that disturbances in one pathway induce other pathogenic mechanisms, resulting in neurodegeneration. Increasing evidence links Golgi fragmentation to recent pathological mechanisms implicated in ALS, including disruption of intracellular trafficking and ER stress. This warrants future studies examining the relationship between Golgi fragmentation to other somatic Golgi functions, including autophagy, and specific neuronal functions of the Golgi, such as axonal homeostasis (Figure 1). The unique characteristics of neurons and the existence of Golgi outposts may confer additional, more specialized functions of the Golgi in these cells which may render these cells more vulnerable to neurodegeneration in ALS.

### Conflict of interest statement

The authors declare that the research was conducted in the absence of any commercial or financial relationships that could be construed as a potential conflict of interest.
